# Supplementary material for: Parameter estimation in a whole-brain network model of epilepsy: Comparison of parallel global optimization solvers
Source: PLoS Comput Biol. 2024 Jul 11;20(7):e1011642. doi: 10.1371/journal.pcbi.1011642 (PMC11265693; doi:10.1371/journal.pcbi.1011642)
Supplement: S1 File — (PDF) [file pcbi.1011642.s001.pdf]

Supporting Information

Parameter estimation in a whole-brain network model of epilepsy: comparison of parallel global optimization solvers

David R. Penas[1,◕], Meysam Hashemi[2,◕], Viktor K. Jirsa[2,*], Julio R. Banga[1,*]

1 Computational Biology Lab, MBG-CSIC (Spanish National Research Council), Pontevedra, Spain.
2 Aix Marseille Univ, INSERM, INS, Institut de Neurosciences des Systèmes, Marseille, France.

◕These authors contributed equally to this work.
* viktor.jirsa@univ-amu.fr , j.r.banga@csic.es

1 The structural connectivity and gain matrix

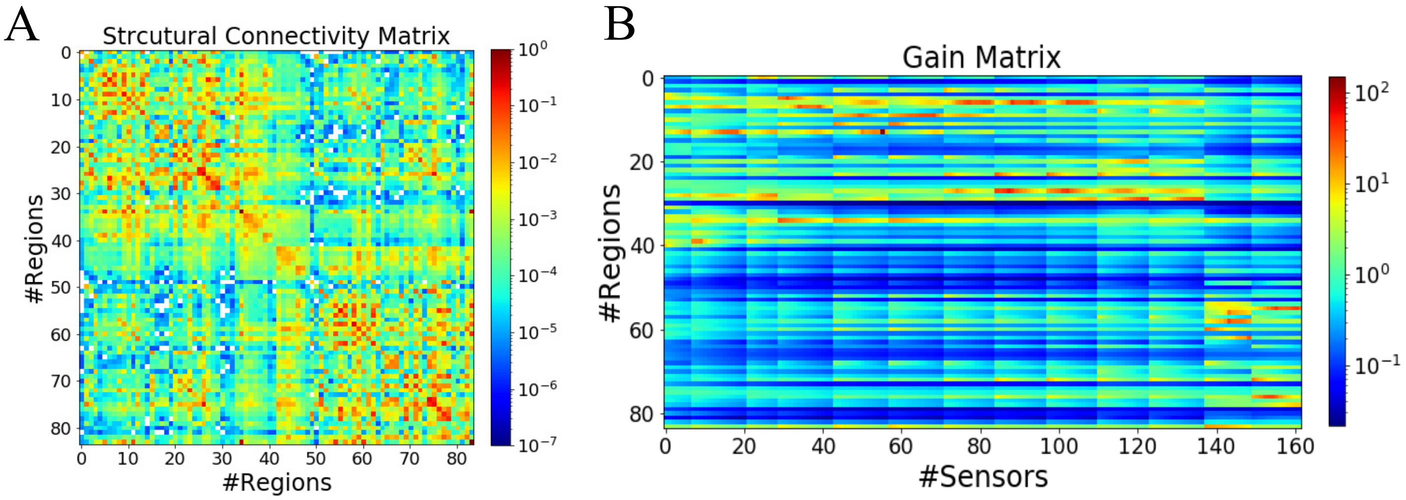

Fig 1. The structural connectivity and gain matrix used in VEP model. (**A**) An exemplary of structural connectivity (SC) matrix, whose entries represent the connection strength between the brain regions, is derived from diffusion MRI tractography. Using Desikan-Killiany parcellation, the brain of the patient is parcellated into 84 different regions comprising 68 cortical regions and 16 subcortical structures. (**B**) Rectangular gain (lead-field or projection) matrix of a randomly selected patient with partial epilepsy. Each element represents the inverse-squared distance between the region and the sensor. By virtue of sparse implantation, most elements of gain matrix are close to zeros.

2 Labels and indices of sub-divided brain regions

Table 1. Labels and indices of sub-divided brain regions

#	label	#	label
01	ctx-lh-bankssts	43	Right-Thalamus-Proper
02	ctx-lh-caudalanteriorcingulate	44	Right-Caudate
03	ctx-lh-caudalmiddlefrontal	45	Right-Putamen
04	ctx-lh-cuneus	46	Right-Pallidum
05	ctx-lh-entorhinal	47	Right-Hippocampus
06	ctx-lh-fusiform	48	Right-Amygdala
07	ctx-lh-inferiorparietal	49	Right-Accumbens-area
08	ctx-lh-inferiortemporal	50	ctx-rh-bankssts
09	ctx-lh-isthmuscingulate	51	ctx-rh-caudalanteriorcingulate
10	ctx-lh-lateraloccipital	52	ctx-rh-caudalmiddlefrontal
11	ctx-lh-lateralorbitofrontal	53	ctx-rh-cuneus
12	ctx-lh-lingual	54	ctx-rh-entorhinal
13	ctx-lh-medialorbitofrontal	55	ctx-rh-fusiform
14	ctx-lh-middletemporal	56	ctx-rh-inferiorparietal
15	ctx-lh-parahippocampal	57	ctx-rh-inferiortemporal
16	ctx-lh-paracentral	58	ctx-rh-isthmuscingulate
17	ctx-lh-parsopercularis	59	ctx-rh-lateraloccipital
18	ctx-lh-parsorbitalis	60	ctx-rh-lateralorbitofrontal
19	ctx-lh-parstriangularis	61	ctx-rh-lingual
20	ctx-lh-pericalcarine	62	ctx-rh-medialorbitofrontal
21	ctx-lh-postcentral	63	ctx-rh-middletemporal
22	ctx-lh-posteriorcingulate	64	ctx-rh-parahippocampal
23	ctx-lh-precentral	65	ctx-rh-paracentral
24	ctx-lh-precuneus	66	ctx-rh-parsopercularis
25	ctx-lh-rostralanteriorcingulate	67	ctx-rh-parsorbitalis
26	ctx-lh-rostralmiddlefrontal	68	ctx-rh-parstriangularis
27	ctx-lh-superiorfrontal	69	ctx-rh-pericalcarine
28	ctx-lh-superiorparietal	70	ctx-rh-postcentral
29	ctx-lh-superiortemporal	71	ctx-rh-posteriorcingulate
30	ctx-lh-supramarginal	72	ctx-rh-precentral
31	ctx-lh-frontalpole	73	ctx-rh-precuneus
32	ctx-lh-temporalpole	74	ctx-rh-rostralanteriorcingulate
33	ctx-lh-transversetemporal	75	ctx-rh-rostralmiddlefrontal
34	ctx-lh-insula	76	ctx-rh-superiorfrontal
35	Left-Cerebellum-Cortex	77	ctx-rh-superiorparietal
36	Left-Thalamus-Proper	78	ctx-rh-superiortemporal
37	Left-Caudate	79	ctx-rh-supramarginal
38	Left-Putamen	80	ctx-rh-frontalpole
39	Left-Pallidum	81	ctx-rh-temporalpole
40	Left-Hippocampus	82	ctx-rh-transversetemporal
41	Left-Amygdala	83	ctx-rh-insula
42	Left-Accumbens-area	84	Right-Cerebellum-Cortex

3 Analyzing the solution of Problem 6

This section analyzes the estimation results in Problem 6, i.e. the stochastic and stiff VEP model at sensor level, characterized by a long seizure envelope due to a large time-scale separation. As stated in the main text, we considered a high value of noise dynamics, represented as zero-mean Gaussian noise with a standard deviation of 0.1, to achieve propagation. The result indicated that, although the system dynamics of regions corresponding to EZ and HZ are accurately estimated, the estimated trajectory for one of the PZs dampens to a stable fixed point, despite the presence of a limit cycle in the true hiddent dynamics.

In this context, we performed further tests to ascertain whether augmenting additional computational resources and refining the initial points could help us retrieve the standard parameters. We also explored a revised cost function for optimization, intending to capture more accurately the stochastic aspect of this issue. Specifically, we adopted the median of 100 simulated RMSE values for each parameter vector as the objective function. Given the computational intensity of this updated cost function, we executed these new tests utilizing SaCeSS with a notably extended time limit (58 hours) and an increased count of parallel processors (64 workers).

Table 2 displays the solutions obtained considering 4 different configurations for these tests. Each reported solution was the best out of 10 runs. The notation **params_true** indicates the nominal value of the parameters used to generate the synthetic data.

Table 2. Solutions obtained by SaCeSS considering four different tests in Problem 6. The 'Initial Guesses' column indicates whether the SaCeSS optimization started from an initial parameter set. The 'Description' column provides a brief explanation of each run.

**solution**	**initial guesses**	**Description**
solution1	random	10 runs were performed using the extra computing resources mentioned previously and optimizing the new cost function based on the median of 100 RMSE values. The parameter values of this solution are represented in red in Figure 2, with their convergence curve also shown in the same color in Figure 4.
solution2	solution1 & **params_true**	We conducted the same experiment as above, but starting from solution1 and **params_true** as initial points. The color blue represents the parameter values of this solution in Figure 2 and its convergence in Figure 4.
solution3	**params_true** perturbed	10 runs were carried out using the new cost function and the extra computational resources, starting from a solution generated by perturbing **params_true** by 10%. Their results, in terms of parameter values and convergence, are presented in orange in Figures 3 and 4.
solution4	**params_true** perturbed	Same than solution 3, but starting from a different solution generated by the perturbation of **params_true** by 10%. Figures 3 and 4 shows their results in green.

Figures 2 and 3 show the parameter values for the solutions mentioned in Table 2. In both graphs, the grey line represents the values of **params_true**. Figure 4 illustrates the convergence of the SaCeSS runs for each test. We note that, even though the initial parameters varied significantly in quality (as indicated by cost function values), nearly all the runs converged to a common final solution in terms of the cost function. Nevertheless, as elaborated further below, these nearly identical solutions in cost do not completely align in terms of estimated parameter values.

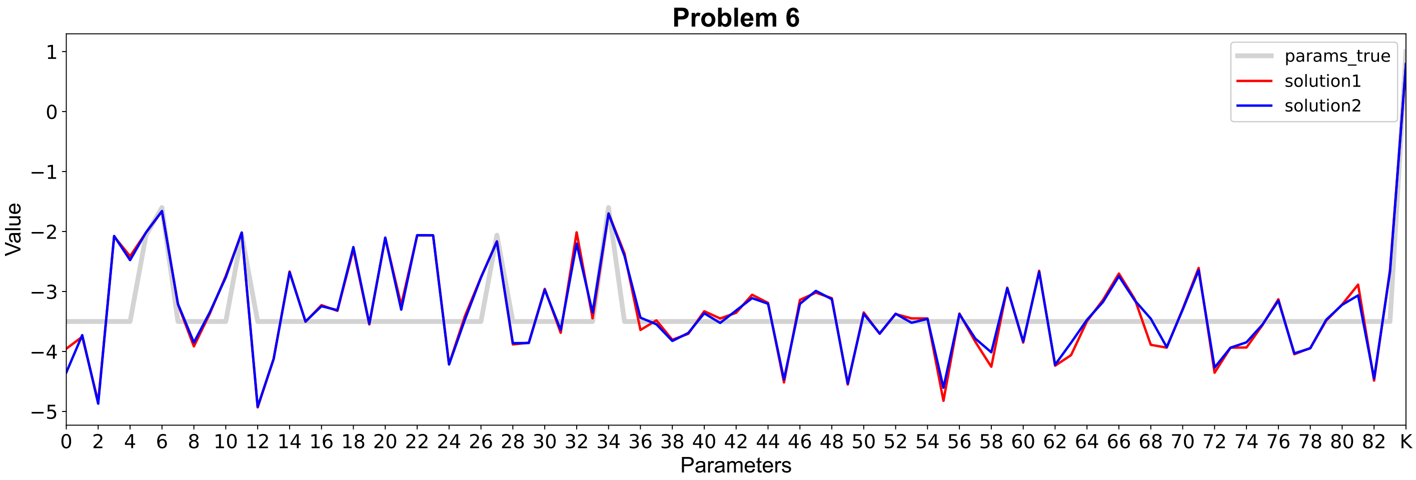

Fig 2. Parallel coordinate plots (I). Representation of parameter values for solution1 and solution2.

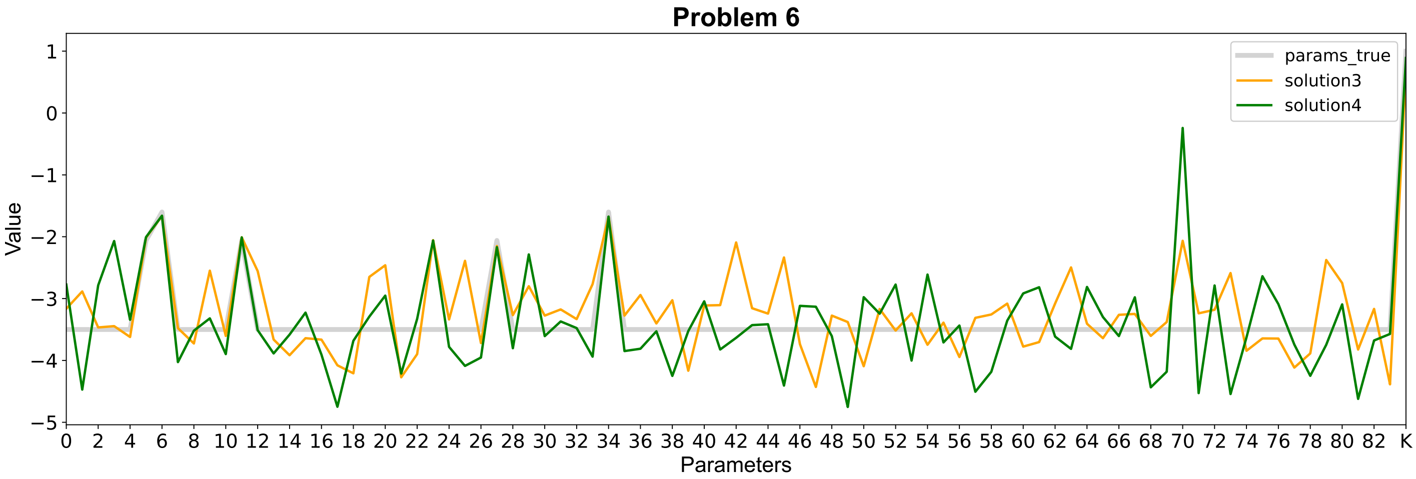

Fig 3. Parallel coordinate plots (II). Representation of parameter values for solution3 and solution4

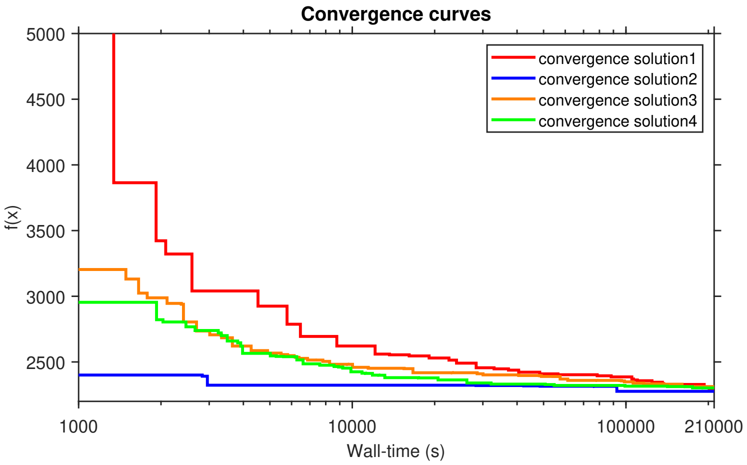

Fig 4. Convergence curves for the different tests considering problem 6. Each curve represents a run of SaCeSS using 64 parallel processors during 58 hours.

With the aim of better comparing the fitness of these different solutions, we conducted a larger set of simulations (1000) to evaluate the cost for each parameter vector, **params_true**, solution1, solution2, solution3, and solution4. Boxplots showing the dispersion of the costs are shown in Figure 5. The median and distributions for the solutions in Table 2 are very similar, with cost function values between 2400-2500. Interestingly, this is significantly lower than the distribution provided by **param_true**, where the median is around 3200.

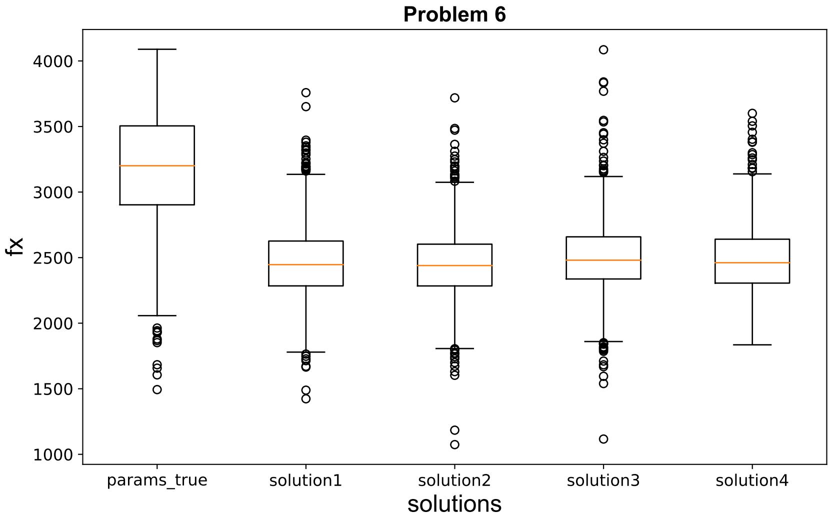

Fig 5. Boxplot comparison of the cost function distribution for the obtained solutions.

Therefore, although the provided solutions exhibit better fitness in terms of the RSME cost function, technically they correspond to a slight overfitting, thus explaining why we could not recover the nominal values of the parameters. However, we also suspect that there is a lack of identifiability caused by the specific cost function considered.

4 Details about hyperparameters tuning with Optuna

One well-known issue with evolutionary algorithms, metaheuristics, and other stochastic methods is that their performance can significantly vary depending on the chosen solver settings (hyperparameters). Moreover, the selection of these hyperparameters is typically problem-dependent.

In this study, we have used Optuna, a Bayesian optimization framework, to develop a methodology for automatically tuning the settings of SaCeSS in order to improve its performance on the VEP benchmarks. It is worth noting that most of the SaCeSS hyperparameters are related to the collaboration and adaptation mechanism among the cooperative workers.

We started by formulating the hyperparameter tuning as a mixed-integer optimization problem with six decision variables representing the SaCeSS configuration options to be tuned:

- **user_options_nstuck (nstuck)**: Maximum number of iterations for a solution in eSS without improvement before generating another solution.
- **local_options_evalmax (evalmax)**: Maximum number of evaluations performed by the local solver.
- **cooperation_evals_threshold (e_thres)**: Parameter to determine whether to reconfigure a slave based on the number of evaluations performed without significantly improving the cooperative solution.
- **cooperation_mult_num_sendSol (mult_num)**: Parameter to determine whether to start the reconfiguration of a slave based on the number of solutions sent by this slave.
- **cooperation_minimum_num_sendSol (min_num)**: Minimum number of received solutions to start the reconfiguration of a slave when such slave has not sent any solution yet.
- **cooperation_threshold_adapt (thr_adapt)**: Value for automatic management of the reception threshold.

Table 4 details the types and ranges of these decision variables. For a more detailed explanation of these hyperparameters, please refer to the SaCeSS solver documentation.

Table 3. Domain of decision variables. This table presents the minimum, maximum, and default values for each variable and its type, which can be integer, float, or categorical (the latter being an integer representing a specific category). The interval column interprets the range of the variables. For example, **evalmax** takes various integer values multiplied by the original problem's dimension.

Parameter	Max	Min	Default	Type	Interval
nstuck	100	10	20	categorical	[10, 20, ..., 90, 100]
evalmax	1000	1	10	integer	[10*D 1000*D]
e_thres	50000	500	5000	categorical	[500, 1000, ..., 49500, 50000]
mult_num	20	5	10	integer	[5 20]
min_num	40	10	20	integer	[10 40]
thr_adapt	20	1	10	float	[1 20]

Next, we formulated this mixed-integer optimization as the minimization a cost function defined by the geometric mean of cost values from n independent SaCeSS runs for a given problem:

minimize:

$$\mathrm{fx} = \left(\prod_{i=1}^{n} \mathrm{best\_cost}_i \right)^{\frac{1}{n}}$$

subject to:

$$\mathrm{best\_cost}_i \leftarrow run_i \text{ SaCeSS } \{\omega, \Phi\}$$

where:

$$\omega \rightarrow \{\text{nstuck, evalmax, e\_thres, mult\_num, min\_num, thr\_adapt}\}$$

$$\Phi \rightarrow 12 \text{ processors during } 30 \text{ seconds}$$

where n is the number of runs (5 in our tests), $\mathrm{best\_cost}_i$ represents the fitness of the best solution reported in run_i, ω is a set containing the hyperparameters of SaCeSS, which serve as the decision variables for the optimization problem, and Φ denotes static computational resource parameters related to the number of workers and the stopping time for each run_i. We have selected this threshold because it is the time required to reach good quality solutions in Problem 1.

As discussed in the main text, obtaining a reasonable solution within a moderate computation time threshold can be challenging in VEP problems. For this reason, we decided to re-estimate all the problems based on the hyperparameter tuning obtained with Optuna considering Problem 1. But even for this problem this is challenging: each evaluation of the objective function involves 5 runs of SaCeSS, this operation will require at least 150 seconds, making the problem quite computationally expensive, so using standard mixed-integer nonlinear solvers would be prohibitive.

Bayesian methods are utilized in optimization when the problem size is not very large and the cost functions are expensive to evaluate, offering reasonable solutions without the need for large number of evaluations. In this context, we chose Optuna because it is widely used in similar contexts, such as in the tuning of hyperparameters of machine learning models. We employed the Bayesian optimization scheme provided by Optuna, using the default algorithm, the Tree-Structured Parzen Estimator (TPE), to determine the best SaCeSS hyperparameters for Problem 1 minimizing the objective function presented above.

Table 4. Hyperparameters calibrated by Optuna in Problem 1.

Parameter	Optuna calibrated settings	SaCeSS default settings
nstuck	20	20
evalmax	921	10
e_thres	13000	5000
mult_num	13	10
min_num	19	20
thr_adapt	9.143	10

The Optuna solution process took 13 hours to complete, and was repeated 10 times to account for the stochastic nature of Bayesian optimization, resulting in the hyperparameters shown in Table 4. These fine-tuned settings are similar to the SaCeSS default values with the exceptions being the maximum number of evaluations for the local solver and the number of evaluations without activity before reconfiguring a worker.

Next, we applied these fine-tuned best settings to the SaCeSS solutions of the other problems. The results were very encouraging, as reported and discussed in the main text. For more details, please refer to the spreadsheet document in the supplementary information available at Zenodo `https://doi.org/10.5281/zenodo.10057789`.
